# Corneal Neovascularization: Pathogenesis, Current Insights and Future Strategies

**DOI:** 10.3390/biology15020136

**Published:** 2026-01-13

**Authors:** Evita Muller, Leo Feinberg, Małgorzata Woronkowicz, Harry W. Roberts

**Affiliations:** 1 West of England Eye Unit, Royal Devon University Healthcare NHS Foundation Trust, Exeter EX2 5DW, UK; evita.muller@nhs.net (E.M.);; 2 Faculty of Health and Life Sciences, University of Exeter Medical School, Exeter EX1 2LU, UK; 3 North Devon District Hospital, Royal Devon University Healthcare NHS Foundation Trust, Barnstaple EX31 4JB, UK; 4 Moorfields Eye Hospital NHS Foundation Trust, London EC1V 2PD, UK

**Keywords:** cornea, corneal neovascularization, CoNV, vascular endothelial growth factor, VEGF, anti-VEGF, angiogenesis, antiangiogenesis, gene-editing, ocular nanosystems, CRISPR

## Abstract

The cornea is an avascular, immune-privileged tissue critical to maintaining transparency, optimal light refraction, and protection from microbial and immunogenic insults. Corneal neovascularization (CoNV) is a pathological sequela of multiple anterior segment diseases and presents a major cause for reduced visual acuity and overall quality of life. Various aetiologies, including infection (e.g., herpes simplex), inflammation (e.g., infective keratitis), hypoxia (e.g., contact lens overuse), degeneration (e.g., chemical burns), and trauma, disrupt the homeostatic avascular microenvironment, triggering an overactive compensatory response. This response is governed by a complex interplay of pro- and anti-angiogenic factors. This review investigates the potential for these mediators to serve as therapeutic targets. Current therapeutic strategies for CoNV encompass topical corticosteroids, anti-VEGF injections, fine-needle diathermy, and laser modalities including argon, photodynamic therapy and Nd:YAG. Emerging therapies involve steroid-sparing immunosuppressants (including cyclosporine and rapamycin), anti-fibrotic agents and advanced drug delivery systems, including ocular nanosystems and viral vectors, to enhance drug bioavailability. Adjunctive therapy to attenuate the protective corneal epithelium prior to target neovascular plexi are further explored. Gene-based approaches, such as Aganirsen (antisense oligonucleotides) and CRISPR/Cas9-mediated VEGF-A editing, have shown promise in preclinical studies for CoNV regression and remission. Given the multifactorial pathophysiology of CoNV, combination therapies targeting multiple molecular pathways may offer improved visual outcomes. Case studies of CoNV highlight the need for multifaceted approaches tailored to patient demographics and underlying ocular diseases. Future research and clinical trials are essential to elucidate optimal therapeutic strategies and explore combination therapies to ensure better management, improved treatment outcomes, and long-term remission of this visually disabling condition.

## Simple Summary

Corneal clarity is maintained by its avascular and immune-privileged status under normal circumstances. Secondary to pathological insult, new immature blood vessels may invade the cornea resulting in profound visual loss and permanent corneal damage. This review article summarises current knowledge on pathological mechanisms by which the neovascularization occurs and its management. This review explores the strengths and limitations of existing treatments, including anti-inflammatory eye drops, injections of agents blocking signals promoting vessel growth, laser treatments and fine-needle diathermy procedures. Novel cutting-edge approaches are highlighted, which involve advanced drug-delivery methods, gene-based therapeutics, and treatments using stem cells or nanoparticles. As corneal neovascularization is a significant cause of preventable vision loss worldwide, novel treatments may prevent disease progression and preserve good visual outcomes in patients. Emphasis is placed on future treatments requiring a combination approach to prove efficient control and cessation of neovascularization.

## 1. Introduction

Corneal neovascularization (CoNV), also known as corneal angiogenesis, is the invasion of new blood and lymphatic vessels over 2 mm from the limbus into the normally avascular corneal tissue [1]. CoNV arises in response to diverse insults—including hypoxia, infection, inflammation, trauma, degeneration or previous surgery—and is a major cause of corneal opacity and vision loss [2,3].

Corneal clarity is sustained by unique corneal angiogenic privilege (CAP), an immune-privileged, angiostatic state maintained by the tightly regulated equilibrium of pro-angiogenic and anti-angiogenic factors. Disruption of CAP promotes vascular endothelial activation, stromal invasion, and development of immature, leaky neovessels that facilitate immune cell trafficking, perpetuating inflammation, oedema, lipid keratopathy, corneal scarring, and reduced visual acuity (VA) [4,5,6,7].

The principal therapeutic aims are to (i) suppress or regress pathological vessels, (ii) restore and preserve corneal clarity, and (iii) reduce corneal graft rejection in patients undergoing keratoplasty.

This review provides a structured, comprehensive overview of both established and emerging therapeutic approaches for the management of CoNV. We first summarise investigative methods and CoNV pathophysiology, followed by a critical appraisal of conventional treatment modalities. Subsequent sections focus on novel and experimental therapeutic strategies, highlighting promising outcomes reported in preclinical models and early clinical studies.

### 1.1. Methodology

This narrative review was informed by a comprehensive search strategy to identify relevant publications focusing on the pathophysiology, current, and future treatment modalities in CoNV. Literature searches were performed using electronic databases, including PubMed, Google Scholar, Web of Science, and Scopus. Search terms encompassed concepts related to the condition itself (“corneal neovascularization,” “corneal angiogenesis,” “limbal neovascularization”), underlying biological mechanisms (“angiogenesis,” “lymphangiogenesis,” “hypoxia,” “inflammation,” “VEGF,” “HIF-1α,” “matrix metalloproteinases,” “cytokines”), and therapeutic approaches. Both established and emerging treatments were included, with search terms such as “anti-VEGF therapy,” “bevacizumab,” “fine needle diathermy,” “laser photocoagulation,” “immunomodulators,” “gene therapy,” “RNA-based therapy,” and “aganirsen.” Boolean operators (AND/OR) were used to combine synonyms and concepts, and truncation was applied where appropriate to maximise sensitivity. No date limits were initially applied to ensure comprehensive retrieval, and reference lists of key articles were hand-searched to identify additional relevant studies.

### 1.2. Epidemiology and Aetiology

Earlier estimated reported approximately 1.4 million cases of CoNV annually in the United States [8], although contemporary population-level prevalence remains uncertain in the context of evolving treatments. Few population-based studies have quantified CoNV prevalence, highlighting the need for updated epidemiological studies in the context of evolving therapeutic modalities.

In 2021, 10.4% of 13,493 patients in an Italian corneal unit were diagnosed with CoNV [9]. Interestingly, these were most commonly non-infectious in aetiology for both unilateral (48.9%) and bilateral (76.8%) cases. Historically, infectious keratitis (IK) has been considered the leading cause of CoNV [10,11,12], with Herpes simplex virus 1 (HSV-1) representing the most common infectious cause of CoNV in the developed world [13,14,15,16,17,18]. Chlamydia trachomatis and onchocerciasis have historically represented major infectious causes of CoNV in endemic regions in the developing world [13,15]. Although several of the epidemiological data informing these associations derive from earlier studies, they remain relevant given the well-characterised and unchanged pathogenic mechanisms underlying these diseases. Moreover, despite substantial progress in public health interventions and disease control, trachoma and onchocerciasis persist in specific endemic populations, and their ocular sequelae continue to be clinically encountered.

Other causes that give rise to CoNV include various forms of infectious (viral, bacterial and, less commonly, acanthamoebic) keratitis [9], chemical injury [13], ocular surface disease including severe dry eye syndrome (secondary to Sjögren syndrome, rosacea, rheumatoid arthritis), ocular cicatricial pemphigoid and Stevens–Johnson syndrome [18], keratoplasty failure and rejection [19,20,21], and hypoxic injury secondary to contact lens overuse [18,22,23].

### 1.3. Pathophysiology

**Figure 1:**
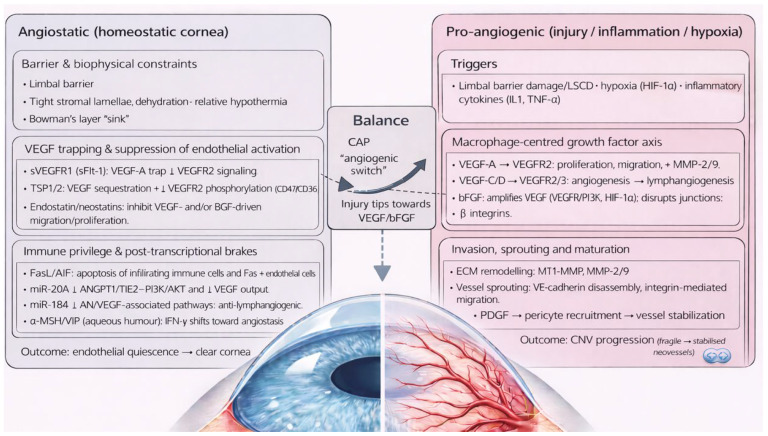
Corneal angiogenic privilege as a complex interplay of angiostatic and pro-angiogenic factors.

CAP is maintained by multiple anti-angiogenic mechanisms that synergistically preserve corneal avascularity. When disrupted by inflammatory or hypoxic injury, the balance shifts towards pro-angiogenic signalling—a transition analogous to the “angiogenic switch” described in tumorigenesis [24], conveyed in Figure 1.

Key anatomical and biochemical anti-angiogenic factors (Table 1) include the limbal barrier, tight stromal collagen lamellae arrangement and dehydration, the corneal relative hypothermic state, possible sequestration of angiogenic factors by Bowman’s layer and anti-angiogenic factors circulating in the aqueous humour, such as α−melanocyte stimulating hormone (α-MSH) and vasoactive intestinal peptide (VIP) [5,11,25,26]. Additionally, soluble and membrane-bound anti-angiogenic factors are constitutively produced throughout the cornea. Soluble vascular endothelial growth factor receptor 1 (sVEGFR1) is a truncated receptor which sequesters vascular endothelial growth factor-A (VEGF-A), competitively inhibiting its action on membrane-bound VEGFR1 [20,27,28]. sVEGFR1 is constitutively expressed in healthy corneal tissue, with reduced levels found in CoNV [29,30].

sVEGFR2 inhibits lymphangiogenesis [31]. Angiostatin is a proteolytic plasminogen fragment produced by corneal epithelial cells that reduces vascular endothelial cell (VEC) proliferation and migration [13]. Constitutive expression of Fas ligand (FasL) [20,32] and Apoptosis-Inducing Factor (AIF) [20,33] bind respective receptors on macrophages, T cells and VECs, inducing apoptosis. Interferon-gamma (IFN-γ) down-regulates transforming growth factor beta- (TGF-β) induced VEGF-A and increases Programmed Death-Ligand 1 (PD-L1) and sVEGFR-1 expression.

Thrombospondin (TSP) -1 and -2 are constitutively expressed, anti-angiogenic signalling glycoproteins within healthy corneal extracellular matrix (ECM). TSP-1 sequesters VEGF, preventing VEGFR2 signalling [34,35]; and reduces VEGF production via binding to CD36 and CD47 in macrophages and VECs, preventing VEGF production and inducing apoptosis, respectively [20,36,37].

Membrane-bound inhibitors of CoNV are also formed by matrix metalloproteinases (MMP)-mediated cleavage of collagen molecules within the corneal ECM. Endostatin and neostatin-7 and -14 are produced by MMP-mediated cleavage of mediated collagen XVIII. Endostatin is the collagen XVIII C-terminal fragment and one of the most potent inhibitors of corneal angiogenesis through inhibition of VEGF- and basic fibroblast growth factor- (bFGF) mediated pathways, in addition to promoting VEC apoptosis through increasing caspase3 activity (an intracellular protease). Endostatin also inhibits lymphangiogenesis through VEGF-C downregulation and promoting apoptosis of VEGFR-3 expressing cells [5,13,18]. Neostatin-7 and -14 are produced by MMP-7 and membrane type-1 MMP (MT1-MMP) cleavage, respectively, and inhibit the bFGF-mediated pathway [5,18]. Arrestin, canstatin, and tumstatin are derived from MMP cleavage of collagen IV and induce VEC apoptosis [5,6,18].

MicroRNAs provide an additional layer of angiostatic regulation at the post-transcriptional level. miR-204, which is highly expressed in the corneal epithelium, inhibits pathological angiogenesis by suppressing ANGPT1/TIE2–PI3K/AKT signalling and reducing VEGF-A expression, thereby limiting endothelial cell survival and proliferation [38,39]. Similarly, miR-184 exerts anti-lymphangiogenic effects by downregulating key pro-angiogenic pathways, including VEGF, Wnt/β-catenin, and Akt signalling, resulting in reduced lymphatic endothelial cell activation [40,41].

CoNV has broadly been divided into two pathways: damage to the limbal stem cell barrier and activation of proinflammatory mediators.

Inflammatory or hypoxic injury to the cornea promotes release of three key angiogenic cytokines: VEGF, bFGF, and platelet-derived growth factor (PDGF). Macrophages are the major source in response to interleukin (IL)-1β, IL-1α and TNFα. Key pro-angiogenic mediators are detailed in Table 2.

VEGF-A plays the dominant role in CoNV through binding VEGFR-1 and VEGFR-2 on the surface of VECs [5,6,42], promoting endothelial proliferation and transmigration [14,43,44,45]. Through VEGFR-2 signalling, VEGF-A upregulates MMP-2 and MMP-9, key factors in ECM remodelling and VEC invasion. Premature neovascular stalks and scaffolding are established, forming dendritic projections, which further release MMPs and facilitate neovascular migration.

Macrophage-derived VEGF-C and VEGF-D promotes angiogenesis through VEGFR-2 [46], and lymphangiogenesis through VEGFR-3 [5,47,48].

bFGF, also known as fibroblast growth factor-2 (FGF-2), is a potent angiogenic factor through several mechanisms: (i) bFGF upregulates VEGF production through ERK- and PI3K-dependent transcriptional activation, HIF-1α stabilisation, VEGF mRNA stabilisation, and induction of VEGF secretion by stromal and inflammatory cells, collectively amplifying the angiogenic response [49,50,51]; (ii) bFGF modulates endothelial adhesion molecules, inducing phosphorylation-mediated disassembly of VEC-cadherin junctions, which enables endothelial sprouting and increasing vascular permeability [32,52,53,54,55]; (iii) bFGF upregulates pro-angiogenic integrins αvβ3 and α5β1, which enhances endothelial adhesion to ECM components, cytoskeletal reorganisation, and directed migration into the corneal stroma [49,56,57]; (iv) bFGF promotes VEC proliferation through MAPK and PI3K [58,59,60]; (v) bFGF promotes vascular destabilisation through ANG2 and MMP release [61,62], particularly MT1-MMP. Macrophage production of macrophage migratory inhibitory factor (MIF) enhances VEC migration and angiogenesis, thereby upregulating pro-angiogenic mediators VEGF and IL-8 [63,64].

Nascent vessels are structurally fragile and leaky due to lack of pericytes, smooth muscle cells, tight junctions and architectural support from the ECM. However, PDGF-mediated recruitment of pericytes and smooth muscle cells stabilises neovessels, rendering them less susceptible to anti-VEGF or anti-bFGF therapies. With 80% of neovessels achieving pericyte coverage within two weeks of development in human corneal models under electron microscopy, resultant neovessels become stabilised to survive independent circulating VEGF [4,65,66]. Therefore, angioregressive therapies targeting VEGF, bFGF or PDGF are most effective when initiated early, before pericyte coverage allows neovessels to persist independently of the angiogenic milieu [67].

Limbal stem cell damage or deficiency (LSCD) represents a second major pathway permitting CoNV [13], and may result from congenital absence, chemical trauma, contact lens overwear or systemic inflammatory disease [68,69]. In vivo studies show CoNV onset and progression following limbal damage [70,71] and regression following limbal stem cell transplantation [72,73].

Neovessel morphology has been characterised by stromal depth, which corresponds closely with the underlying aetiology. Deep CoNV overlying Descemet’s membrane is seen in herpetic and luetic interstitial keratitis; stromal CoNV, in stromal keratitis; and fibrovascular pannus—the most common pattern—arises from perilimbal fibrovascular proliferation and invasion in ocular surface disorders [13,74,75]. Faraj et al. propose a classification according to vessel maturity (active young, active old mature, partially regressed and regressed), which carries therapeutic relevance [12]. Angioregressive treatments are most effective for active young vessels rather than mature vessels, whereas fine needle diathermy achieved greater efficacy in active, old, and mature vessels.

### 1.4. Clinical Assessment

Clinical assessment of CoNV is essential for guiding treatment selection, monitoring response, and stratifying graft rejection risk. Slit-lamp biomicroscopy remains the primary assessment tool, enabling evaluation of vessel morphology, quadrant involvement, depth, and associated stromal changes such as oedema, lipid keratopathy, or scarring [2]. However, slit-lamp examination is subjective and limited in detecting deep or quiescent vessels.

Standardised slit-lamp photography allows longitudinal documentation and semi-quantitative analysis of neovascular area and vessel density [2]. Fluorescein angiography (FA) provides functional assessment of vessel perfusion and leakage, distinguishing active from mature vasculature, while indocyanine green angiography (ICGA) improves visualisation of deep stromal and afferent feeder vessels and facilitates targeted vessel-occlusive procedures [12,76]. Anterior segment optical coherence tomography and OCT-angiography offer non-invasive, depth-resolved visualisation and quantitative assessment of corneal vascular networks [77].

## 2. Current Treatments

Current treatment strategies for CoNV (Table 3) focus on the suppression of inflammatory and angiogenic signalling rather than elimination of established vessels. First-line therapies include topical corticosteroids and non-steroidal anti-inflammatory drugs, which reduce inflammation-driven angiogenic stimuli but are limited by variable efficacy and ocular side effects. Anti-vascular endothelial growth factor (anti-VEGF) agents, administered topically, subconjunctivally, or intrastromally, provide targeted inhibition of neovascular growth and are most effective against immature vessels, although responses are often incomplete and transient. Immunomodulatory agents such as ciclosporine, tacrolimus, sirolimus, and everolimus attenuate T-cell-mediated inflammation and downstream pro-angiogenic cytokine release, offering steroid-sparing control in chronic inflammatory states associated with CoNV. In parallel, matrix metalloproteinase (MMP) inhibitors limit extracellular matrix degradation and endothelial migration, thereby modulating vessel invasion into the cornea. Procedural adjuncts, including laser photocoagulation, fine-needle diathermy, and corneal cross-linking, may be employed to ablate or stabilise neovessels, though durability and safety profiles vary.

### 2.1. Corticosteroids

Inflammation is a central driver of CoNV, shifting the cornea from its normally anti-angiogenic, immune-privileged state toward a pro-angiogenic milieu [78].

Corticosteroids exhibit potent anti-inflammatory action through inhibition of the arachidonic acid pathway via phospholipase A2, downregulating prostaglandin production [79,80,81]. Systemic administration down-regulates IL-1, IL-3, IL-6, IL-8 and indirectly IL-2 production, dampening T-lymphocyte activation and VEGF gene expression in vascular smooth muscle cells [82]. However, corticosteroids carry significant adverse effects, including ocular hypertension, glaucoma [83], cataract formation [84], and secondary infection [85]. Prolonged systemic use may cause metabolic, psychiatric, and endocrine disturbances, increase infection risk, and precipitate peptic ulceration, thromboembolic events, and growth suppression in children [86,87,88].

### 2.2. Non-Steroidal Anti-Inflammatory Drugs

Non-steroidal anti-inflammatory drugs (NSAIDs) inhibit COX-mediated prostaglandin synthesis. Systemic COX-2 inhibition suppresses VEGF- and bFGF-induced angiogenesis, with greater potency against bFGF-driven pathways [89]. Topical bromfenac reduces COX-2, VEGF, MCP-1 expression [90] where bromfenac nanopolymers provide up to 48 h sustained anti-angiogenic activity while effectively inhibiting CoNV in vivo [91]. While mitigating the long-term adverse effects of corticosteroids, topical NSAIDs carry risks of epithelial toxicity—including epithelial defects, sterile infiltrates and corneal melt [92,93,94]—which limit their clinical utility.

Current guidance does not recommend NSAIDs as first-line therapy for CoNV, though function as adjuncts to suppress early inflammation preceding neovascularization [95]. Combined delivery systems, such as diclofenac with bevacizumab in a subconjunctival thermosensitive hydrogel, further reduce VEGF expression and CoNV with sustained release and favourable biocompatibility [96].

### 2.3. Anti-VEGF Agents

Anti-VEGF agents have become an important therapeutic option for CoNV by targeting the central VEGF-mediated pathway [97]. Bevacizumab (BCZ), ranibizumab (RBZ) and aflibercept (AFL) inhibit VEGF-A–mediated VEC proliferation, migration, and vascular permeability, and promote regression of immature neovessels [98,99,100,101,102,103]. While their use in CoNV is off-licence, they have become an important therapeutic option for immature CoNV of early onset. Other novel monoclonal antibodies include pan-VEGF agent brolucizumab [104] and dual-pathway inhibitor faricimab [105]. Although both agents have demonstrated potent anti-VEGF activity in posterior segment neovascular disease models and are clinically indicated for wet-age related macular degeneration, diabetic macular oedema and retinal vein occlusion [106], there are currently no published preclinical evaluations of these agents in experimental corneal neovascularization models to date. Their effects in this context remain unexplored.

#### 2.3.1. Bevacizumab

Bevacizumab (BCZ) is an IgG1 antibody targeting VEGF-A isoforms [107] and remains the most common off-licence anti-VEGF agent to treat CoNV, owing to its widespread clinical accessibility and cost-effectiveness [24]. BCZ was first FDA approved for use in colorectal cancer in the mid-2000s and was soon adopted off-label as an intravitreal injection in treatment of choroidal neovascularization secondary to wet age-related macular degeneration, diabetic macular oedema and retinal vein occlusions [107].

a. Topical

Prospective clinical studies support the efficacy of topical BCZ for CoNV, reducing the neovascular area by 47–66%, and vessel calibre by 24–54%, although corresponding improvement in visual acuity (VA) was inconsistent [108,109,110,111,112]. Topical BCZ effectively regressed neovessels in high-risk penetrating keratoplasty in three patients with no graft rejection episodes between 12 and 36 months.

While higher concentrations (2.5%) appear well tolerated [112], epithelial toxicity remains a concern [108,109]. Kim et al. reported epithelial defects or erosions in 6/10 eyes, including one case of corneal thinning, all in patients with prior epithelial defects [111]. Dastjerdi et al. also described a case of spontaneous corneal perforation five months after initiating therapy in a patient with a pre-existing epithelial defect [109]. Caution has been advised with courses extending beyond one month and in patients with significant pre-treatment epitheliopathy [111]. No systemic or allergic adverse events were reported, even with prolonged use [108]. Current evidence is limited by small, heterogeneous cohorts, highlighting the need for larger, standardised prospective trials.

b. Subconjunctival

Prospective evidence indicates that subconjunctival BCZ has therapeutic benefit primarily in early disease onset. A randomised trial of monthly subconjunctival BCZ injections reduced neovascularization by 36% in patients with CoNV onset under 6 months [113], and marked regression of CoNV formation within one month of chemical injury [114]. A randomised double-blinded phase III trial of 38 patients found subconjunctival BCZ was only superior to placebo in a sub-analysis of patients with CoNV onset of less than one year, though without statistical significance, suggesting diminishing responsiveness as vessels mature [115]. Chu et al. reported CoNV recurrence in a third of patients six months following treatment cessation, with variable response to repeated injections, suggesting irreversible PDGF-mediated vessel maturation and stabilisation, limiting anti-VEGF therapeutic effect [116].

Evidence for subconjunctival BCZ supporting graft survival has been variable. A prospective interventional case series reported reduced CoNV, maintained graft clarity, and graft survival 9 months after BCZ [117], while a multicenter randomised controlled trial of 92 patients found no significant difference between subconjunctival BCZ and placebo in endothelial rejection in high-risk keratoplasty [118].

Although animal models show higher stromal BCZ concentrations and greater neovessel regression with subconjunctival versus topical therapy [119,120], a meta-analysis demonstrated a greater reduction in CoNV area following topical BCZ administration (48%) compared with subconjunctival injections (32%) [121]. Further standardised trials are required to clarify optimal timing, route and clinical indications.

c. Intrastromal

Intrastromal BCZ has been investigated in enhancing graft survival, particularly in mature or deep CoNV. In high-risk graft candidates, intrastromal BCZ produced complete regression in 14% of high-risk graft candidates, obviating the need for corneal transplantation, while 57% of patients who proceeded to keratoplasty experienced no cases graft rejections or CoNV recurrence during three-year follow up [122]. Intrastromal delivery may achieve higher drug concentrations within the deep stroma than subconjunctival or topical administration [123], and may be considered for deep or treatment-resistant CoNV. Moreover, intrastromal BCZ was found to be safe and well-tolerated, with no significant ocular or systemic adverse effects [124].

#### 2.3.2. Ranibizumab

Ranibizumab (RBZ) is a 48 kDa recombinant humanised monoclonal antibody fragment derived from BCZ with high affinity binding to VEGF-A [78,95]. Owing to the absent Fc region and smaller molecular size (approximately one third of BCZ), it is considered to have better corneal penetration than BCZ [3,78].

Subconjunctival RBZ has been shown to reduce VEGF levels across all anterior segment tissues, including the cornea, iris, aqueous humour and conjunctiva [125], while topical RBZ reduced mean neovascular area by 55% in a small, open-label, non-comparative series at 16 weeks [67].

However, RBZ’s greater affinity to VEGF-A and greater tissue penetration has not clearly conferred clinical superiority over BCZ. A study of 29 eyes treated topically with RBZ or BCZ showed significant reduction in neovascular area and vessel calibre without showing an intergroup statistical difference [11]. A study of 16 eyes showed a single subconjunctival/intrastromal BCZ injection achieved a significantly lower mean neovascular area than eyes treated with subconjunctival/intrastromal RBZ (28% versus 4.5%) [126].

Comparative advantages for BCZ include its longer half-life (approximately 20 days versus six hours for RBZ); greater bioavailability due to reduced tissue clearance; lower cost (RBZ is approximately 10 to 30-fold more expensive than BCZ), and broader clinical evidence base for topical and subconjunctival use in CoNV. However, existing studies are small, heterogeneous with no randomised controlled trials directly comparing agents.

#### 2.3.3. Aflibercept

Aflibercept (AFL) is a soluble decoy fusion receptor consisting of the human IgG1 Fc domain fused to the extracellular ligand-binding domains of VEGFR-1 and VEGFR-2. It has significantly higher VEGF sequestration compared with BCZ and RBZ owing to its 100-fold greater affinity, in addition to placental growth factor (PlGF), conferring dual anti-angiogenic property [78,127,128,129].

Preclinical studies of AFL for CoNV, however, show mixed efficacy. While AFL inhibited FGF-2 mediated neovascularization refractory to BCZ and RBZ in one murine model [130], others have reported no significant differences between AFL and BCZ or RBZ with topical and subconjunctival administrations in murine and rabbit models, respectively [103,131].

Comparisons of subconjunctival AFL with subconjunctival betamethasone or combination therapy of AFL and betamethasone in rabbit models were similarly inconclusive [132]. Sella et al. initially demonstrated superior efficacy in murine CoNV regression with topical AFL compared to BCZ [133]; however, a subsequent prospective clinical trial was terminated early due to lack of therapeutic benefit [134]. Topical AFL was reported to regress CoNV in exposure keratopathy in an isolated paediatric case [135]; however, no further human studies have since investigated its use.

#### 2.3.4. Tocilizumab

Tocilizumab (TCZ) is a humanised monoclonal antibody targeting IL-6 receptor with increasing utility across a number of autoimmune conditions. Preclinical animal model studies have demonstrated anti-angiogenic potential in CoNV. The suture-induced animal models are commonly used to mimic chronic, persistent inflammatory CoNV [136], whereas the alkali burn models represent an acute assault-driven neovascular response [137]. In a rabbit suture model, subconjunctival TCZ significantly reduced CoNV area comparable to BCZ with reductions in VEGF and IL-6 [138]. Similarly, topical IL-6R blockade inhibited CoNV in a murine model of alkali chemical injury [139]. TCZ has also shown improved keratoplasty survival in a rat allograft model, likely through Th17/T regulatory cell modulation and reducing VEGF expression [140]. Current evidence therefore positions tocilizumab as an experimental agent with potential utility in inflammatory or VEGF-driven CoNV but requires formal toxicology studies and early-phase clinical trials before therapeutic application can be considered.

### 2.4. Fine Needle Diathermy

Direct occlusion of corneal vessels using fine needle diathermy (FND) emerged in the early 2000s as an effective and low-cost method for treating established CoNV and became widely adopted for the treatment of mature stromal neovascular complexes resistant to medical therapy [141]. The original description and early clinical series demonstrated that FND reliably occluded neovessels and could be repeated, when necessary, but was associated with occasional intrastromal bleeding and crystalline deposits. Subsequent development of an electrolysis-based needle provided comparable effectiveness, with improved versatility and flexibility, becoming the preferred approach in many centres [142].

Long-term clinical data support FND efficacy in CoNV. In a 5-year case series, 68% demonstrated CoNV regression at first follow-up, 89% after one or two treatments [143]. FND reduced lipid deposition in 82.3% of 17 eyes and achieved the best results in mature vessels [12]. Retreatment (2–5 times) was required in a third of eyes.

Neoadjuvant FND has been used pre-keratoplasty to reduce recipient bed vascularity to improve graft survival. Faraj et al. reported 85% keratoplasty 12-month survival following FND in eyes at high-risk of graft rejection [12]. Combination therapy may enhance outcomes. FND combined with topical and/or subconjunctival BCZ was superior to BCZ monotherapy in CoNV regression prior to high-risk keratoplasty [144]. A study of pre-treatment with FND and subconjunctival BCZ in 22 high-risk keratoplasties reported 73% graft survival at three-year follow-up [145]. FND monotherapy may upregulate several VEGF growth factors and macrophage infiltration, and some authors have recommended FND to be performed in conjunction with topical anti-angiogenic agents to mitigate rebound neovascularization [146].

Angiographically guided, selective FND can optimise vessel targeting and reduce collateral tissue injury. One to three episodes of FND targeting smaller afferent vessels visualised with fluorescein and indocyanine green angiography reduced neovascular area in eyes refractory to topical steroid [147].

FND remains an effective, low-cost, and generally safe method for occlusion of established corneal vessels [12,78]. Faraj et al. report reversible intrastromal haemorrhage in 42% cases [78]. Other reversible complications include striae and whitening. Microperforation is a rare but potentially severe complication on passage of the diathermy needle. Corneal thinning and stromal scarring are rare longer term complications [12]. Cautious, selective use of FND is recommended given incomplete understanding of long-term sequelae to the corneal endothelium and limbal stem cells [75,147].

### 2.5. Laser Therapy

#### 2.5.1. Argon

Argon laser photocoagulation was first described in the early 1970s as a method to induce remission of CoNV in rabbit models [148]. Argon energy is selectively absorbed by haemoglobin molecules, generating thermal coagulation, thrombosis, and vessel regression. Photocoagulation with Argon laser and 577 nm yellow-light regressed CoNV in high-risk patients pre-keratoplasty and with lipid keratopathy [149,150].

With the advent of newer laser systems offering greater tissue specificity and safety profiles, argon lasers are no longer considered the preferred modality. Reported complications including thermal injury to the corneal endothelium, crystalline lens, suture lysis, and corneal thinning. Temporary/reversible reported complications include iris atrophy and pupil ectasia [141,150]. Afferent vessels are less responsive to argon given their deeper stromal location, high vascular flow rates, and are more prone to reopening, requiring multiple treatments [147]. Wider, more superficial efferent vessels with slower blood flow are therefore the recommended target of photocoagulation [78]. More recent case reports have highlighted combination therapy of laser with subconjunctival BCZ as a potential therapeutic strategy [151,152].

#### 2.5.2. Nd:YAG

Prospective studies have demonstrated effective Nd:YAG ablation of CoNV. In a cohort treated with frequency doubled Nd:YAG (533 nm), complete vessel occlusion was achieved in 54% of vessels, 9% showed partial occlusion, and 37% recanalized three months after treatment [153]. Adverse events were reported in 43% of patients, most commonly iris damage and corneal haemorrhage, which spontaneously resolved after one week. Other complications include corneal thinning, iris atrophy, and crystalline deposition [150,154]. Whilst Nd:YAG ablation has therapeutic potential, multimodal treatment strategies are recommended to minimise progression.

#### 2.5.3. Femtosecond

Femtosecond laser systems operate in the near-infrared spectrum and induce photodisruption through ultrashort pulses, causing less collateral damage than Nd:YAG. Owing to its 106-fold shorter pulse duration, femtosecond laser has been approved for cataract and corneal refractive surgery [155,156].

Femtosecond laser reduced CoNV by 30% after five days of successive treatment in murine models and its low photodisruptive energy threshold may offer a minimally invasive treatment option [157]. However, paucity of human clinical studies and high costs limit its availability, where femtosecond laser remains logistically inaccessible compared with conventional laser modalities.

#### 2.5.4. Photodynamic Therapy

Photodynamic therapy (PDT) with verteporfin has been licenced for treatment of central serous chorioretinopathy. Verteporfin photosensitization by PDT forms free radicals and reactive oxygen species on VEC membranes, leading to vasoconstriction, thrombosis and aberrant vessel occlusion [158]. Early case reports and small series using PDT for CoNV-lipid keratopathy offered promising results with favourable safety profiles [159,160,161]. In a prospective series of 18 eyes treated with PDT, CoNV regressed in 77.8%, with complete vascular occlusion in 50% and improved visual acuity of ≥2 lines in 38.9% at 12 months, with recurrence in 11.1% [162]. Al-Torbak observed CoNV regression in two-thirds of 33 eyes [163]. Both series noted lower therapeutic effect with increasing CoNV area and neovessel depth. Adverse effects were rare, indicating minimal collateral tissue injury.

Combination therapy may confer additional benefit: PDT with subconjunctival BCZ demonstrated greater vessel regression compared to PDT-monotherapy [164]. However, verteporfin-PDT is limited by its cost, lengthy treatment protocols, and ongoing global verteporfin shortages [165]. Additionally, current evidence is limited to small, uncontrolled case series.

### 2.6. Immunomodulators

Immunomodulatory agents block key enzymes and molecular signalling pathways in the adaptive immune response. Calcineurin inhibitors are potent T cell inhibitors, targeting the calcium-dependent phosphatase enzyme central to T cell activation and clonal T cell expansion.

#### 2.6.1. Cyclosporine

Proposed mechanisms for cyclosporine’s anti-angiogenic effect include HESR1 overexpression and VEGFR2 downregulation [166]. Cyclosporin A (CsA) has been shown to inhibit VEGF-mediated VEC migration in vitro [167].

Animal studies reported reduced graft rejection and CoNV regression with topical or systemic CsA: topical CsA outperformed BCZ but remained less effective than dexamethasone. Clinical evidence is limited, however subconjunctival CsA implants have shown minimal effect in reducing neovascularization in high-risk grafts [168], likely due to inadequate stromal penetration relative to systemic administration [169]. Given its significant adverse effect profile, including renal dysfunction [170], systemic CsA is not recommended for preventing graft rejection nor CoNV regression. CsA-loaded nanofibers, however, have demonstrated greater anti-inflammatory and anti-angiogenic effect than topical formulations, highlighting the potential for optimised local drug delivery systems [171].

#### 2.6.2. Tacrolimus

Tacrolimus exerts both T and B cell immunomodulatory effects [172] and is widely used in preventing solid organ allograft rejection and several autoimmune diseases. Its anti-angiogenic effect is mediated through suppression of key pro-angiogenic factors—including EGF, FGF, PDGF, histamine, prostaglandins, TNF-α, MMPs and IL-1 and IL-6 [173]—and provides comparable immunosuppression to cyclosporin at far lower concentrations [174].

Preclinical studies consistently demonstrate significant CoNV regression with systemic and topical tacrolimus [175]. Topical and subconjunctival tacrolimus showed higher neovascular regression than subconjunctival BCZ [176,177]. Limited available clinical evidence supports sustained remission of herpetic CoNV following topical tacrolimus taper. In high-risk keratoplasty, topical tacrolimus reduced rejection rates compared with cyclosporine (16% vs. 46%), with more favourable adverse effect profile [178]. Tacrolimus ointment appears well tolerated, although increased incidence of dry eye symptoms and risk of ocular surface infection are reported [178].

#### 2.6.3. Sirolimus and Everolimus

Sirolimus (rapamycin) and everolimus (its derivative) are mammalian target of rapamycin (mTOR) inhibitors used in unresectable metastatic perivascular epithelioid tumours, renal transplant rejection prophylaxis [179,180,181,182] and topical use in facial angiofibromas [183]. Both suppress cell growth, migration, and angiogenesis through mTOR signalling and inhibit T cell cycle progression from G1 to S phase [184].

Preclinical studies consistently demonstrate that topical and systemic sirolimus reduce CoNV through the downregulation of IL-6, TGFb1, and HIF-1a/VEGF-mediated pathways, along with broader suppression of pro-angiogenic cytokines [185,186]. Comparative evidence suggests everolimus outperforms topical sunitinib, achieving greater reduction in VEGFR-2 and ERK1/2 [187].

In summary, immunomodulatory agents may be valuable steroid-sparing agents in the CoNV armamentarium. However, with limited clinical studies, poorly understood toxicity profile and therapeutic index in human corneal tissue, their application remains restricted to animal models.

### 2.7. Anti-Matrix Metalloproteinases

MMPs are zinc-containing endopeptidases central to ECM degradation [188], enabling VEC migration and release of other angiogenic factors including IL-1, IL-6, VEGF, and bFGF [189,190,191,192,193]. MMP upregulation precedes CoNV, and MMP-2-deficiency attenuates CoNV in experimental models [194].

Doxycycline, a tetracycline with potent MMP-inhibitory and anti-inflammatory activity, reduces macrophage infiltration, cytokine expression and VEGF-C/VEGFR-3 signalling in murine corneas [195,196,197]. Oral doxycycline showed greater CoNV inhibition than oral and topical dexamethasone, with faster epithelial wound healing [195].

Limited clinical data report partial regression with topical tetracyclines, including 50% regression with 1% doxycycline in six patients [198] and 71% with a topical tetracycline gel over four months [199]. Newer tetracyclines such as tigecycline also significantly suppress CoNV in rodents [200], and subconjunctival doxycycline appears safe in experimental doses [201].

Given the multifactorial nature of CoNV, tetracyclines may be most effective as adjuncts, particularly in combination with anti-VEGF or corticosteroid therapy [202,203]. Larger controlled clinical trials, including combination regimens, are needed to define their therapeutic role.

## 3. Future Therapies

Future therapeutic strategies for CoNV (summarised in Table 4) aim to overcome limitations of current anti-inflammatory and anti-VEGF approaches, particularly limited durability, resistance in mature vessels, and the need for repeated administration. Emerging modalities seek to enhance treatment longevity, target multiple angiogenic and fibrotic pathways simultaneously, and improve ocular bioavailability while minimising toxicity.

### 3.1. Receptor Tyrosine Kinase Inhibitors

Receptor tyrosine kinase inhibitors (RTKIs) are a rapidly expanding class of molecular drug agents used in oncology and other systemic conditions [204], and represent a promising next-generation pharmacological strategy for CoNV by simultaneously targeting multiple pro-angiogenic signalling pathways. Unlike anti-VEGF monotherapy blockade of a single ligand, RTKIs block multiple pro-angiogenic receptors—including VEGFRs, PDGFRs and FDFGRs—inhibiting multiple downstream signalling pathways driving VEC proliferation, migration, and survival [205]. Given the expanding range of RTKIs where the majority of current evidence is derived from preclinical models, with the exception of an early phase prospective study for pazopanib, well-designed clinical non-inferiority trials are required to evaluate their efficacy and safety against established anti-VEGF therapies.

#### 3.1.1. Pazopanib

Pazopanib is a multi-target tyrosine kinase inhibitor (TKI) that inhibits VEGFR-1–3, PDGFR, and FGFR [206], and is FDA-approved for treatment of renal cell carcinoma and soft-tissue sarcoma [207]. Emerging evidence also suggests early promise for pazopanib in the management of CoNV. Its proposed clinical role is to provide broader angiogenic pathway suppression with topical delivery, potentially reducing treatment frequency.

In a prospective study of 20 patients, three weeks of topical pazopanib significantly reduced CoNV area and vessel length at 12 weeks [208]. Punctal plugs were introduced to limit systemic absorption, as oral pazopanib has been reported to cause hypertension, deranged liver and thyroid function tests. No systemic side effects were reported, and visual acuities remained stable. However, the evidence remains limited, and the absence of randomised or comparative trials precludes firm conclusions regarding its clinical utility.

#### 3.1.2. Lapatinib

Lapatinib is an RTKI inhibiting HER2/EGFR signalling, VEGF expression and MMP-mediated ECM remodelling, and is used in conjunction with capecitabine in refractory HER2-positive breast cancer [209]. Its clinical rationale in CoNV is the suppression of angiogenesis and stromal invasion through non-VEGF mechanisms. Oral lapatinib in a rat model tended towards superiority over oral trastuzumab in inhibiting CoNV [210]. In the absence of further studies evaluating lapatinib ocular pharmacokinetics and safety profile, its therapeutic potential in CoNV remains undetermined.

#### 3.1.3. Regorafenib

Regorafenib was approved for use in metastatic colorectal cancer and gastrointestinal stromal tumours, and subsequently for hepatocellular carcinoma in 2017 [211]. A murine model of CoNV did not show superior efficacy of topical regorafenib compared with BCZ or dexamethasone [212]. No further studies have examined regorafenib utility for CoNV.

#### 3.1.4. Sunitinib

Sunitinib has shown the most compelling preclinical efficacy among RTKIs for CoNV and is proposed as a means to inhibit both angiogenic sprouting and vessel maturation. Sunitinib is a multi-kinase RTKI blocking VEGFR-2, PDGFR-β, FGFR-1, and EGFR [95]. In comparative studies on rabbit eyes, topical sunitinib reduced CoNV by 82%, compared to 28% with topical BCZ [66]. Furthermore, sunitinib inhibited VEGF and PDGF pathways approximately threefold more effectively than BCZ. Subsequent animal studies obtained similar results comparing sunitinib to BCZ [213,214]. Moreover, topical sunitinib showed superior efficacy to subconjunctival injection in a rabbit model [213]. More recently, combination therapy of sunitinib with hesperetin—a citrus-derived flavonoid with vasoprotective properties—produced a 97% CoNV reduction in rat models, exceeding sunitinib monotherapy and sunitinib-doxycycline combination [215].

Emerging research in animal models has focused on more precise and innovative sunitinib delivery using lipid and polymeric nanocarriers [216] as well as biodegradable microspheres [217]. However, sunitinib remains an experimental modality without established translational applicability for CoNV. Although its multi-target angiogenetic inhibition provides a strong mechanistic rationale, the current evidence base is insufficient to support its integration into clinical practice. Robust translational studies and well-designed early-phase clinical trials are necessary to define its potential therapeutic role in CoNV.

#### 3.1.5. Axitinib

Axitinib is a novel RTKI with profound inhibition of CoNV in rabbit models. Topical axitinib inhibited up to 85% neovascularized area, with effective blockade of VEGF and PDGF signalling across all concentrations [218]. However, axitinib exhibits higher binding affinity for TIE2 than sunitinib or vorolanib, potentially disrupting a receptor pathway essential for vascular stability and anti-inflammatory signalling [219]. This interaction may limit its suitability to inhibit CoNV development in animal models relative to sunitinib.

#### 3.1.6. SU6668

SU6668 is a small-molecule multi-target tyrosine kinase inhibitor blocking VEGFR-2, PDGFR-β, and FGFR-1 with anti-angiogenic potential [220,221] developed as an anti-angiogenic agent. A recent rat corneal suture model showed topically delivered nanoparticle formulations of SU6668 administered twice daily regressed CoNV area over days with favourable safety profile [222]. An additional study incorporating SU6668 into complex theranostic nanoparticle systems combined with photothermal therapy showed targeted accumulation and photothermal closure of neovessels, and sustained anti-angiogenic suppression via SU6668 release, with proposed synergy through HSP70 downregulation [223]. The evidence base for SU6668 is entirely pre-clinical, limited to acute injury models, small cohorts of animal models with short follow-up and unknown long-term ocular safety.

### 3.2. Antifibrotic Agents

Antifibrotic therapies aim to address stromal scarring and fibrosis, which often coexist with CoNV and contribute to irreversible visual loss even after vessel regression.

#### 3.2.1. Losartan

Losartan is an angiotensin II receptor antagonist anti-hypertensive agent with potential antifibrotic mechanisms through inhibition of TGF-β-mediated ECM and collagen deposition [224]. Rabbit models suggest losartan may additionally reduce fibroblast-myofibroblast differentiation and expression of alpha-smooth muscle (α-SMA) [225]. Emerging evidence suggests losartan monotherapy [226] and in combination with scleral lenses [227] may reverse stromal haze and fibrosis may in human corneas. With a low toxicity profile at doses of less than 0.8 mg/mL [228], losartan represents a promising adjunct to reduce CoNV-induced fibrosis and warrants further research [229].

#### 3.2.2. Decorin

Decorin is a small leucine-rich proteoglycan which has emerged as an antifibrotic and anti-angiogenic therapeutic target in CoNV [230]. Although decorin can facilitate angiogenesis by mediating endothelial cell adhesion to collagen I and α1β2 integrin [231], it also exhibits anti-angiogenic property depending on the tissue microenvironment, including suppression of VEGF expression in tumour models [232,233]. Decorin is also a potent inhibitor of TGF-β, fibroblast-myofibroblast differentiation, and subsequent α-SMA expression [95].

In vivo studies on rabbit models found decorin gene therapy delivered through adeno-associated viral (AAV) serotype 5 significantly reduced CoNV area, length and calibre, accompanied by downregulation of pro-angiogenic genes and upregulation of anti-angiogenic pathways, with sustained efficacy and favourable safety over six months [234]. Decorin-deficient mice also exhibited more extensive CoNV, likely due to dysregulated VEGF expression [235].

As an endogenous molecule with an encouraging preclinical safety profile, decorin represents a promising therapeutic candidate. However, further studies are required to clarify its dualistic pro- and anti-angiogenic functions, and clinical applicability in human CoNV.

#### 3.2.3. Pirfenidone and Nintedanib

Pirfenidone is an anti-inflammatory and antifibrotic agent used to treat idiopathic pulmonary fibrosis (IPF) [236] and has demonstrated reduced corneal antifibrotic activity through vitamin E-loaded contact lenses [237] and liposomal nanosystems [238], likely modulating TGF-β-mediated fibroblast–myofibroblast differentiation and collagen deposition [239,240,241,242]. Additionally, intravitreal pirfenidone downregulated VEGF in murine models, reducing choroidal neovascular leakage and lesions sizes [243].

Nintedanib, also licenced for IPF, may be more immediately translatable to CoNV. As a VEGFR-2 TKI, topical and systemic nintedanib has demonstrated anti-inflammatory effects and inhibited lymphangiogenesis [244]. In rabbit models, topical nintedanib demonstrated superior efficacy to topical BCZ, possibly through downregulating P38 MAPK and AKT signalling pathways [245]. Thermosensitive-hydrogel formulations improve corneal penetration, producing marked inhibition and sustained remission in murine studies [246,247].

### 3.3. Antioxidants and Redox-Modulating Therapies

Oxidative stress is an important upstream contributor to CoNV across multiple aetiologies [97,248]. Excess reactive oxygen species (ROS) generated within the corneal epithelium and stroma amplify inflammatory signalling cascades, promote leukocyte recruitment, and enhance transcription of pro-angiogenic mediators such as VEGF and bFGF [249]. Accordingly, antioxidant and redox-modulating therapies have been proposed as adjunctive strategies aimed at attenuating ROS-driven inflammatory amplification, limiting permissive angiogenic signalling, and supporting epithelial and stromal recovery, rather than directly inducing regression of established neovessels [250].

Mechanistically, ROS excess in CoNV activates redox-sensitive pathways, including NF-κB, MAPK and hypoxia-responsive signalling, which upregulate cytokines (IL-1, IL-6, TNF-α), matrix metalloproteinases and angiogenic growth factors [249,251]. Antioxidant approaches therefore seek to interrupt early pathogenic triggers that sustain the angiogenic switch, particularly in inflammation-dominant and injury-related CoNV, and may complement anti-VEGF or immunomodulatory therapies by reducing recurrence and treatment burden [250].

#### 3.3.1. Vitamin E (α-Tocopherol)

Vitamin E has received particular attention due to its dual role as a lipid-soluble antioxidant and as a functional excipient capable of prolonging ocular surface drug residence. In a rabbit alkali burn model, vitamin E–loaded contact lenses used to deliver pirfenidone significantly increased ocular drug bioavailability and improved corneal inflammatory and fibrotic outcomes compared with topical drops, supporting the feasibility of antioxidant-enabled sustained delivery platforms in CoNV-relevant chemical injury states [237].

#### 3.3.2. Coenzyme Q10

Coenzyme Q10 (ubiquinone) is a mitochondrial redox cofactor with antioxidant and cytoprotective properties. In a suture-induced rabbit model of CoNV, subconjunctival administration of a combined coenzyme Q10 and vitamin E preparation resulted in a reduction in neovascular area compared with untreated controls [252]. However, bevacizumab achieved greater neovascular regression, underscoring that antioxidant-based therapy is unlikely to match the efficacy of direct VEGF blockade in active disease. Current evidence remains limited to small preclinical or pilot studies, with uncertain dosing optimisation and unclear relative contribution of individual antioxidant components.

#### 3.3.3. Gallic Acid, Epigallocatechin Gallate, and Related Polyphenols

Polyphenolic antioxidants, including gallic acid and epigallocatechin gallate (EGCG), have attracted interest due to their combined antioxidant and anti-inflammatory effects with secondary modulation of angiogenic signalling. Topical EGCG inhibits corneal neovascularisation in rabbit models, likely via suppression of inflammatory mediators and endothelial activation rather than direct endothelial cytotoxicity [253]. More recently, nano-engineered platforms incorporating EGCG and gallic acid have demonstrated marked experimental suppression of CoNV, supporting the potential of combined antioxidant–anti-angiogenic strategies [254]. However, current evidence remains largely preclinical, with heterogeneity in models and formulations; limited ocular bioavailability and penetration constrain translation.

Overall, antioxidant and redox-modulating therapies are best viewed as adjuncts in CoNV, with greatest relevance in early or inflammation-dominant disease and post-injury prophylaxis. Their value lies in dampening oxidative–inflammatory amplification, supporting epithelial repair and potentially improving durability of anti-angiogenic or immunomodulatory treatments; however, current evidence does not support antioxidants as stand-alone therapies for established or mature CoNV, underscoring the need for translational studies with robust clinical endpoints.

### 3.4. Ocular Nanosystems

Ocular nanosystems aim to improve drug delivery, bioavailability and treatment durability, reducing dosing frequency and toxicity associated with conventional eye drops.

Nanotechnology is a novel, rapidly progressing field aimed at enhancing drug delivery and bioavailability through nanoscale organic materials. Ocular nanosystems may overcome limitations of current therapeutic agents through optimised drug delivery, enhanced bioavailability, longer therapeutic window and localization at the nanometre scale [255]. Nanosystems offers several therapeutic avenues for CoNV, through improved drug delivery with nanocarriers, as therapeutic nano-agents, or as vectors for targeted gene therapy [10].

A variety of nanoscale platforms have been engineered for ocular delivery, including liposomes, nanoparticles, polymeric micelles, nanoemulsions, dendrimers and nanowafers [256,257]. Owing to the scope of this review, it was not possible to discuss all nanosystems currently under investigation for CoNV. Accordingly, for clarity and conciseness, this review selectively highlights two nanosystems supported by substantial preclinical evidence that demonstrate the greatest potential for clinical applicability and near-term translation.

#### 3.4.1. Nanoemulsions and Microemulsions

Nanoemulsions and microemulsions differ in structure, stability, droplet size and thermodynamics. Nanoemulsions contain emulsifiers and coemulsions with droplets ranging from 20 to 200 nanometers, whilst microemulsions have surfactant and cosurfactants with smaller droplets at 10–100 nanometers [258]. Both enhance drug delivery by increasing ocular surface bioavailability with sustained drug release. Nanoemulsions are kinetically stable and mix nanodroplets with precorneal constituents, facilitating wider drug distribution across the ocular surface. By contrast, microemulsions are thermodynamically stable and have superior intraocular penetration and bioavailability.

Experimental nanoemulsions incorporating naringenin [259], Ro5-3335 [260], isoliquiritigenin [261], and sunitinib [262] have inhibited CoNV secondary to chemical alkali-induced animal burn models [263]. Further studies are required across broader aetiologies and in human models [264].

#### 3.4.2. Nanowafers

Nanowafers are thin, transparent discs composed of drug-loaded individual nanoresevoirs [261]. Similar to contact lenses with daily application to the corneal surface, nanowafers withstand blinking pressures and dissolve following sustained, gradual release ranging from hours to days, increasing bioavailability and absorption compared to conventional eye drops [263].

Axitinib-eluting nanowafers have demonstrated marked anti-angiogenic activity in murine models of CoNV: daily nanowafer application was twice as effective as twice-daily topical axitinib eye drops [265].

Subsequent studies report antifibrotic effects and improved wound healing with dexamethasone drug-eluting nanowafers, where daily application provided similar effectiveness as QID topical dexamethasone eye drops [266]. Moreover, drug-free dextran-sulfate polymer nanowafers inhibited scarring, neovascularisation and fibrosis in murine corneas, suggesting a biomaterial-mediated therapeutic effect independent of pharmacological agents [267]. This drug-delivery nanowafer may improve patient compliance and reduce adverse responses to conventional eye drops.

Nonetheless, nanowafer-based therapy for CoNV remains preclinical and experimental, with long-term safety, human efficacy, cost-effectiveness, and toxicity still undetermined [263]. Further translational and clinical studies are required to define its role alongside current anti-inflammatory and anti-angiogenic treatments.

### 3.5. Corneal Crosslinking

Corneal crosslinking (CXL) is an established method of care for preventing progression of corneal ectasia such as keratoconus, by induction of stromal collagen crosslinking through riboflavin photoactivation with ultraviolet-A (UVA) irradiation [268,269,270] and stabilising corneal biomechanics [271]. More recently, CXL has been adopted off-label for IK and postoperative ectasia management [272,273,274,275,276].

Emerging evidence suggests that CXL may exert anti-angiogenic effects. Murine models demonstrated CXL transiently reduced angiogenic mRNA and protein expression [277], reduced trauma-induced CoNV and improved corneal wound healing [278].

CXL may provide a potential neoadjuvant treatment option in high-risk keratoplasty. In prevascularized murine corneas, pre-operative CXL prolonged graft survival and reduced macrophage and CD45+ cell infiltration [279]. Combined CXL-FND reduced CoNV and inflammatory cell recruitment compared with either as monotherapy [280]. Early clinical data supports these findings: neoadjuvant peripheral CXL prior to penetrating keratoplasty reduced CoNV by 70%, without graft revascularization or rejection episodes over four month follow-up [281]. A multicenter, randomised controlled trial is underway to investigate graft outcomes following CXL preconditioning [282].

Alternative irradiation strategies, including ruthenium- and blue-light-based protocols, have demonstrated significant inhibition of corneal and limbal neovascularization in vivo with potentially improved biocompatibility compared to conventional UVA regimens [283].

Nevertheless, CXL carries a theoretical risk of herpes simplex keratitis reactivation and recurrent CoNV in predisposed eyes, warranting long-term follow-up in high-risk patients [284]. Overall, CXL represents a promising neoadjuvant strategy for mitigating CoNV in revascularized corneas and improving outcomes in high-risk keratoplasty. Further optimisation of irradiation protocols and confirmation through large-scale clinical trials are needed to establish its role as an anti-angiogenic therapy.

### 3.6. Mitomycin C Intravascular Chemoembolization

Mitomycin-C intravascular chemoembolization (MICE) directly targets mature, VEGF-independent neovessels, addressing a key limitation of pharmacological therapy, with targeted injection directly into corneal neovessels.

MMC is an antineoplastic alkylating agent that induces DNA cross-linking and cell-cycle arrest, resulting in targeted cytotoxicity. Delivered intravascularly, MICE directly occludes both afferent and efferent corneal vessels through injection of the primary feeder vessels, with no adverse events reported to date [285]. Unlike anti-VEGF therapies, MICE can target mature neovessels but is best suited to larger calibre, identifiable feeder vessels rather than diffuse neovascularization.

Emerging clinical evidence suggests MICE may be effective for both treatment and prophylaxis. A case report demonstrated long-term remission of CoNV one year after prophylactic use prior to penetrating keratoplasty [286]. A prospective study of eight patients demonstrated complete remission at one-year median follow-up with no adverse effects [287].

MICE represents a promising emerging therapeutic modality for CoNV. As MMC use in this context remains off-licence, larger controlled studies are required to establish long-term safety, efficacy and optimal dosing.

### 3.7. Gene Therapy

Gene-based approaches aim to provide long-term or permanent suppression of angiogenic signalling, reducing reliance on repeated pharmacological intervention.

Emerging gene-based therapies are redefining approaches to pathological angiogenesis, offering alternatives to conventional anti-VEGF agents and immunomodulators [288,289]. Gene therapy delivers modified genetic material to modulate expression of disease-targeted genes.

Gene transfection into host cells can occur via intrastromal, subconjunctival injections, electroporation, and gene guns. Targeting VEGF expression remains the predominant strategy in CoNV. Subconjunctival administration of plasmids encoding GA-binding protein—an inhibitor of VEGF and roundabout-4 transcription—produced transient but measurable anti-angiogenic effects in experimental CoNV [290].

#### 3.7.1. Aganirsen and Antisense Oligonucleotides

Aganirsen targets insulin-receptor substrate-1 (IRS-1) and has demonstrated efficacy in phase III trials, reducing CoNV and transplant requirement. Despite orphan drug designation, commercial availability remains limited.

Antisense oligonucleotides (ASO) are short single stranded DNA or RNA sequences designed to bind complementary targeted pro-angiogenic mRNA sequences upregulated in CoNV. IRS-1 is one overexpressed mediator, with a critical role in CoNV initiation and progression [291]. Aganirsen, a novel IRS-1–targeting ASO, has demonstrated dose-dependent suppression of IRS-1 expression and inhibition of pathological neovascularization in vitro. In a multicenter phase III study (I-CAN), aganirsen significantly reduced CoNV and lowered the proportion of patients requiring corneal transplantation, with favourable safety and tolerability profile [292]. Aganirsen has subsequently received orphan drug designation in the United States for prevention of corneal graft failure, though it remains commercially unavailable at present. Further long-term studies are warranted to confirm durability of effect and fully establish its clinical safety and utility.

#### 3.7.2. CRISPR-Cas 9

Clustered Regularly Interspaced Short Palindromic Repeats (CRISPR)-Cas 9-mediated VEGF-A disruption offers durable anti-angiogenic effects in animal models but faces challenges related to delivery, off-target effects, and immune responses.

CRISPR-Cas 9 genome-editing system has emerged as a highly versatile and cost-effective platform for therapeutic gene modification, with numerous clinical trials underway for ocular and systemic diseases [293].

CRISPR-Cas 9-mediated targeting of VEGF-A in CoNV has demonstrated promising results. In vitro gene editing resulted in substantial VEGF-A depletion, suppressing endothelial cell proliferation and migration. In vivo, subconjunctival delivery of a Cas9–AAV dual-vector system targeting VEGF-A effectively inhibited suture-induced CoNV in murine corneas [294]. These findings suggest that CRISPR-Cas 9-induced VEGF-A frameshift mutation may offer a long-term therapeutic strategy for refractory CoNV.

Emerging CRISPR/Cas strategies for ocular gene editing, including those with potential application to CoNV, face several delivery and safety-related challenges. Efficient in vivo delivery of CRISPR components to corneal cells remains limited by anatomical barriers and vector constraints; viral vectors such as AAV, while effective, have restricted packaging capacity and carry risks of vector-associated immune activation, whereas non-viral systems often achieve lower editing efficiency [295].

Pre-existing immunity to Cas proteins and innate immune responses to delivery vehicles can provoke local inflammation and reduce therapeutic durability, even in the immune-privileged cornea [296]. Off-target genomic alterations and the need for temporal control of editing activity are additional safety concerns that must be addressed before clinical translation [296,297].

#### 3.7.3. Viral Vectors

Adenovirus-associated viruses (AAV) and lentivirus-mediated expression of anti-angiogenic genes has shown sustained efficacy in preclinical models, though high costs and regulatory hurdles currently limit clinical translation.

Introduction of anti-angiogenic genes into host cell DNA may occur via transfection of viral DNA with the encoded desired gene through viruses as vectors. These commonly include adenovirus, lentivirus, or retroviruses. Viral vectors demonstrate the most efficient gene transfer rates at 80% [298] and although primarily developed for monogenic retinal diseases, these systems have been explored for ocular angiogenesis.

AAV vectors expressing endostatin, angiostatin, or soluble Flk-1 significantly reduced CoNV in murine models [299,300], with similar results in rabbit models using lentivirus-based vectors [301]. AAV-mediated expression of soluble Flk-1 receptors also reduces CoNV by competitively binding VEGF [302]. More recently, single stromal injection of AAV-delivered conbercept achieved sustained anti-VEGF activity for over three months with favourable safety [303].

AAV application remains experimental. Concerns exist regarding theoretical pathogenic activation, insertional mutagenesis and systemic exposure, although available evidence suggests that the genotoxic and tumorigenic risks of AAV are low [304,305]. Clinical translation is limited by high development costs, insufficiently defined safety and efficacy profiles, preventing progression to larger scale clinical trials.

### 3.8. Stem Cell Therapy

Stem cell-based therapies seek to restore corneal homeostasis and suppress inflammatory-mediated angiogenesis, rather than directly targeting angiogenic ligands.

Mesenchymal stem cells (MSCs), MSC-derived extracellular vesicles (MSC-EVs), and induced pluripotent stem cells (iPSCs) represent emerging regenerative strategies demonstrated anti-angiogenic and regenerative potential in preclinical and early clinical settings. However, issues related to safety, standardisation, and long-term outcomes remain significant barriers to widespread clinical adoption.

MSCs are multipotent mesodermal cells capable of differentiating into chondrocytes, adipocytes, and osteoblasts [306], with immunomodulatory and anti-inflammatory action. Their secretome can be delivered via MSC-EVs to transport cytokines, growth factors, lipids, and mRNAs involved in signalling pathways [307,308]. iPSCs, produced by reprogramming somatic cells with Yamanaka factors, retain pluripotent differentiation potential similar to embryonic stem cells. iPSCs may give rise to corneal-relevant cell types, including keratocytes and corneal epithelial-like cells. Early first-in-human clinical studies suggest that iPSC-derived cells may contribute to secretion of anti-angiogenic and immunoregulatory factors in treatment of limbal stem cell deficiency [309,310] and bullous keratopathy [311]. However, challenges related to control of differentiation, tumorigenicity and scalable clinical translation remain to be elucidated.

#### 3.8.1. Mesenchymal Stem Cells

Given their presence in the corneal limbus, MSCs have become a major focus in CoNV [312,313]. Allogeneic MSCs can evade immunosurveillance and integrate host tissue without rejection [314]. Systemic MSC slowed CoNV progression in mice by decreasing macrophage recruitment and VEGF-C and VEGF-D levels [315].

Corneal-derived MSCs (cMSCs) displayed anti-angiogenic potential in both in vitro and in vivo models through PEDF and sFLT-1 production [316] and modulation of macrophage phenotypes [317]. In rabbit alkali-burn models, intrastromal and subconjunctival MSCs improved re-epithelialization and CoNV [318].

#### 3.8.2. Route of Administration

Optimal approach for MSC-based therapy delivery in CoNV remains under investigation. Subconjunctival injection has demonstrated superior corneal wound healing and VEGF expression than amniotic membrane-based MSC delivery [319]. Adipose-derived MSCs reduced CoNV onset and progression [320,321], with enhanced efficacy when MSCs are administered promptly after injury via combined topical, intrastromal and subconjunctival routes [320].

Topical MSCs derived from orbital fat also offer a non-invasive route that supports corneal epithelial healing and tissue regeneration; however, they do not appear to alter TNF-α, TGF-β, or VEGF levels, and showed limited direct anti-angiogenic effect [322]. Collectively, these findings highlight both the therapeutic potential and complexity of MSC-based interventions for CoNV.

#### 3.8.3. Extracellular Vesicles and Beyond

Topical, cell-free therapies such as MSC-EV drops may avoid complications arising from whole stem cell therapy, reducing the risk of rejection, unregulated cell proliferation, tumorigenesis and toxicity. As carriers of MSC anti-angiogenic factors, MSC-EVs provide a promising route for direct corneal delivery [323].

Bone-marrow-derived MSC-EVs reduced CoNV in murine models [324], although a rabbit model of MSC-EV (exosome) treatment did not significantly reduce CoNV but improved corneal clarity and stromal haze [325].

More recent ultrathin amniotic membrane stroma inoculated with MSCs simulated healthy corneal epithelium, producing a transparent, anti-angiogenic scaffold mimicking the limbal stem cell microenvironment [326].

#### 3.8.4. Summary of Mesenchymal Stem Cells

No clinical studies have directly evaluated MSCs for CoNV treatment. Preliminary evidence indicates that intrastromal MSC delivery improved visual outcomes in advanced keratoconus [327], and early-phase studies have demonstrated the safety and feasibility of MSC-based therapies for limbal stem cell deficiency [328,329], primary and graft-versus-host associated dry eye disease [330,331], and persistent epithelial defects [332]. These emerging data highlight growing interest in MSC therapies for corneal disease and may inform future translation to CoNV [333].

MSC therapies have also been associated with severe adverse outcomes, including ocular hypertension, haemorrhagic retinopathy, vitreous haemorrhage, retinal detachment, lens dislocation, and irreversible vision loss, largely linked to unregulated, direct-to-consumer stem cell interventions [334,335].

Before MSC-based therapies can be safely evaluated in large clinical cohorts, key challenges must be addressed. These include clarifying the mechanisms and pharmacological properties of MSCs and MSC-EVs, such as bioavailability and tissue distribution, and establishing standardised, regulated protocols for MSC isolation, culture, and differentiation [308]. 

## 4. Conclusions

CoNV represents a significant clinical challenge due to its multifactorial pathophysiology, propensity for visual impairment, and variable response to conventional therapies. Current treatment options—ranging from corticosteroids and anti-VEGF agents to laser-based interventions and fine-needle diathermy—provide important symptomatic and structural control but often fail to achieve durable remission, particularly in cases driven by chronic inflammation or recurrent insults. Advances in drug-delivery technologies, including nanosystems and viral vectors, offer improved tissue penetration and sustained delivery, enhancing therapeutic efficacy while reducing treatment burden.

Mesenchymal stem cell (MSC)-based therapies and MSC-derived extracellular vesicles (MSC-EVs) have emerged as promising candidates for ocular surface regeneration due to their immunomodulatory, anti-inflammatory, and anti-angiogenic properties. Early-phase clinical studies in dry eye disease have demonstrated the safety and feasibility of MSC delivery using well-regulated protocols. Conversely, reports of severe adverse outcomes associated with unregulated, direct-to-consumer stem cell interventions underscore the critical need for rigorous oversight, standardised manufacturing practices and robust clinical trial validation.

The breadth of evidence across both established and emerging therapies highlights the necessity for multimodal treatment strategies that target the complex interplay of angiogenic, inflammatory and fibrotic pathways in CoNV. Integrating molecularly targeted agents, regenerative therapies, and optimised delivery systems may ultimately offer more effective and sustained control of this vision-threatening condition.

### Future Directions

Future progress in the management of CoNV will depend on addressing several key scientific and translational challenges. A major priority is the continued elucidation of the mechanisms of MSCs and MSC-EVs, particularly their pharmacological properties, biodistribution, and interactions with resident corneal cell populations. A deeper understanding of these mechanisms will facilitate optimisation of dosing strategies, delivery routes and safety profiles.

Parallel to mechanistic studies, there is a pressing need for standardised, regulated protocols governing MSC isolation, expansion and differentiation to mitigate variability and ensure consistent therapeutic quality. Such harmonisation is essential for advancing legitimate clinical applications while safeguarding against the risks highlighted by adverse outcomes associated with unregulated, direct-to-consumer stem-cell interventions.

Therapeutically, the field is moving toward the development of targeted and combinatorial approaches that more effectively address the multifactorial nature of CoNV. Integrating anti-angiogenic, anti-inflammatory, and regenerative modalities may yield synergistic benefits, particularly for refractory disease. Advances in drug-delivery technologies—including nanoparticle-based systems, sustained-release platforms, hydrogels, and emerging gene-editing strategies such as CRISPR-Cas 9-mediated VEGF-A modulation—have the potential to greatly enhance tissue penetration, bioavailability, and therapeutic durability. Improving adjunctive strategies, such as methods for transiently modifying or removing the corneal epithelium to increase access to pathological vascular networks, may further enhance the efficacy of targeted therapies.

Finally, the translation of these innovations into clinical practice will require robust validation through large-scale, long-term, randomised clinical trials, complemented by real-world evidence capturing diverse patient populations and disease phenotypes. Ethical oversight and patient education will also remain central to preventing misuse of stem-cell technologies and maintaining trust in emerging treatments. Collectively, these avenues of research and regulatory development will be critical for achieving sustained, safe, and effective therapeutic outcomes for patients with CoNV.

## Figures and Tables

**Table 1:** Anti-angiogenic mechanisms maintaining corneal angiogenic privilege.

	FACTOR	PRIMARY SOURCE/LOCATION	MECHANISM
Barrier AND biophysical constraints	Limbal barrier	--	Physical/functional boundary restricting vascular ingrowth from limbal/conjunctival vessels.
	Tight stromal collagen lamellae; stromal dehydration	--	Dense, highly ordered ECM and low hydration reduce permissiveness for endothelial invasion (limits migration tracks and sprout extension).
	Relative corneal hypothermia	--	Lower temperature suppresses enzymatic and signalling kinetics that support angiogenesis.
	Bowman’s layer (putative “sink”)	--	Proposed sequestration/compartmentalisation of pro-angiogenic factors, limiting diffusion to responsive stromal endothelium.
VEGF ligand trapping (dominant angiostatic axis)	sVEGFR1	Corneal epithelium & stroma	Binds VEGF-A with high affinity, functioning as an endogenous VEGF-A trap; prevents VEGF-A binding membrane VEGFRs (particularly VEGFR2-driven angiogenic signalling).
	sVEGFR2	Corneal epithelium & stroma	Soluble decoy activity preferentially restricts lymphangiogenic signalling.
Immune-modulatory soluble inhibitors	α-MSH; VIP	Aqueous humour	Anti-inflammatory neuropeptides that dampen inflammatory activation (thereby indirectly suppressing induction of VEGF/bFGF/PDGF in injury contexts.
	IFN-γ	T-lymphocytes, NK cells	Shifts tissue state toward angiostasis by suppressing VEGF induction programs and increasing immune checkpoint/angiostatic factors (e.g., PD-L1; sVEGFR1).
ECM-associated angiostatic signalling	TSP-1	Epithelial basement membrane and ECM	Multifunctional inhibitor: (i) binds VEGF and suppresses VEGFR2 phosphorylation/signalling; (ii) ligates CD47 (and CD36) to inhibit endothelial responses and promote apoptosis (caspase-linked programmes in remodelling endothelium).
	TSP-2	Epithelial basement membrane and ECM	Constitutive ECM angiostatic glycoprotein; complements TSP1-mediated suppression of endothelial activation.
Proteolytic ECM fragments (“matrikines”)	Endostatin	Collagen XVIII C-terminal fragment	Potent inhibitor of corneal angiogenesis and lymphangiogenesis: inhibits VEGF- and bFGF-driven angiogenic programmes, promotes VEC apoptosis through Caspase3 activity.
	Neostatin-7/-14	Collagen XVIII fragments; MMP-7 and membrane type 1 -MMP cleavage, respectively	Selectively antagonise bFGF-induced corneal NV, dampening bFGF-dependent endothelial migration and downstream matrix-remodelling programmes.
	Arrestin, canstatin, tumstatin	MMP cleavage of Collagen IV	Antiangiogenic matrikines, promote VEC apoptosis.
Immune privilege–linked apoptosis	Fas ligand (FasL)	Corneal epithelium/endothelial cells	Engagement of Fas on infiltrating inflammatory cells and Fas+ VECs triggers apoptosis, limiting inflammatory amplification and VEC proliferation.
	AIF	VEC, macrophages	Pro-apoptotic signalling contributing to deletion of pro-angiogenic inflammatory and endothelial effector cells.
Post-transcriptional angiostatic control	miR-204	Corneal epithelium	Limits pathological angiogenesis by repressing ANGPT1/TIE2–PI3K/AKT survival signalling and reducing VEGF pathway output (reported decreases in ANGPT1/VEGF axis in corneal NV models).
	miR-184	Corneal epithelium	Angiostatic/anti-lymphangiogenic microRNA; suppresses key pro-angiogenic signalling modules including Akt and VEGF-associated pathways; reduces lymphatic endothelial activation in corneal lymphangiogenesis models.

**Table 2:** Pro-angiogenic mechanisms driving corneal neovascularization.

CLASS	FACTOR/PROCESS	PRIMARY SOURCE/TRIGGER	MECHANISM (EXPANDED)
Initiation (“angiogenic switch”)	Inflammatory or hypoxic injury	Infection, trauma, hypoxia	Induces a tissue state shift toward pro-angiogenic transcription and secretion (HIF-linked VEGF induction; cytokine-driven growth factor release).
	Limbal stem cell damage/deficiency	Chemical injury, contact lens overwear, systemic inflammatory/autoimmune disease, congenital, iatrogenic/surgical trauma	Removes the limbal “gatekeeper,” enabling vessel entry and sustained surface inflammation that maintains VEGF/bFGF/PDGF production.
Upstream inflammatory drivers	IL-1α, IL-1β, TNF-α	Activated macrophages	Induce macrophage and stromal production of VEGF, bFGF and PDGF; amplify leukocyte recruitment and endothelial activation (per your text).
Core angiogenic ligands	VEGF-A	Macrophages, neutrophils, dendritic cells; stressed epithelial and endothelial cells, stromal keratocytes	Binding VEGFR2 activates pro-angiogenic signalling (MAPK/ERK and PI3K/AKT survival; permeability and migration programmes) and induces MMP-2/MMP-9, enabling stromal invasion and sprout extension.
	VEGF-C/VEGF-D	Macrophages	Promote blood vessel growth (VEGFR2) and lymphangiogenesis (VEGFR3), expanding the vascular/immune trafficking network.
bFGF as an amplifier AND “sprouting enabler”	bFGF (FGF-2)	Macrophages, stressed epithelial and endothelial cells, stromal keratocytes	Multimodal pro-angiogenic driver: (i) increases VEGF output via ERK/PI3K-dependent transcription and HIF-1α stabilisation; (ii) disrupts endothelial junctional integrity (VE-cadherin complex destabilisation); (iii) induces pro-migratory integrins (αvβ3, α5β1); (iv) promotes MT1-MMP/MMP axis activation supporting ECM invasion; (v) stimulates endothelial proliferation via MAPK/PI3K signalling.
Matrix remodelling (permissive stromal invasion)	MMP-2, MMP-9	VEGF-activated VECs; macrophages	Degrades stromal ECM, creates migration paths, and remodels BM barriers; facilitates “tip/stalk” advance and dendritic projections releasing more proteases.
	MT1-MMP (MMP14)	Endothelial cells/fibroblasts	Supports bFGF-driven programmes and promotes stromal invasion; integrates with Ras–ERK pathways in bFGF→VEGF-A upregulation within corneal fibroblasts.
Endothelial activation modules	VE-cadherin junction disassembly	bFGF-driven phosphorylation events	Weakens adherens junctions → endothelial sprouting and increased permeability (leaky nascent vessels).
	Integrins αvβ3 and α5β1	Endothelial surface	Promote adhesion to ECM, cytoskeletal remodelling, and directional migration into stroma; associated with ocular surface angiogenesis contexts.
Macrophage-mediated reinforcement	MIF (macrophage migratory inhibitory factor)	Macrophages	Enhances endothelial migration and sustains a pro-angiogenic cytokine milieu (including VEGF/IL-8 as in your text).
Vessel maturation AND persistence (therapy-relevant)	PDGF (PDGF-B/PDGFRβ axis)	Inflammatory/stromal signals	Recruits pericytes and smooth muscle cells → neovessel stabilisation, reduced leakiness, and reduced responsiveness to anti-VEGF monotherapy; substantial pericyte coverage occurs early in human corneal NV.

**Table 3:** Current pharmacological and procedural therapies for CoNV: key properties, advantages, and limitations.

Therapy/Agent	Class	Molecular Size/Modality	Route (s)	Relative Cost	Key Advantages	Key Limitations/Adverse Effects
Corticosteroids (e.g., dexamethasone, prednisolone)	Anti-inflammatory	~390–430 Da	Topical, subconjunctival, systemic	Low	Potent suppression of IL-1, IL-6, TNF-α; rapid onset	Ocular hypertension, glaucoma, cataract, infection; unsuitable long term
NSAIDs (e.g., bromfenac, diclofenac)	Anti-inflammatory	~250–350 Da	Topical, systemic	Low–moderate	COX inhibition; suppresses prostaglandin-driven VEGF/bFGF; steroid-sparing	Epithelial toxicity, corneal melt; limited monotherapy efficacy
Cyclosporine A	Immunomodulator	1202 Da	Topical, systemic	Moderate	T-cell suppression; inhibits VEGF-mediated migration	Limited stromal penetration; renal and systemic toxicity
Tacrolimus	Immunomodulator	804 Da	Topical, systemic	Moderate	Potent steroid-sparing; suppresses VEGF, PDGF, IL-1, IL-6, TNF-α	Infection risk; ocular surface irritation
Sirolimus/Everolimus	Immunomodulator (mTOR inhibitors)	~914/~958 Da	Topical, systemic	High	Suppresses VEGF, HIF-1α, IL-6; anti-angiogenic and immunosuppressive	Limited human data; corneal toxicity profile unclear
Tocilizumab	IL-6R antibody	~148 kDa	Topical (experimental), subconjunctival	High	Targets IL-6–VEGF axis; potential steroid-sparing	Experimental for CoNV; no established dosing
Bevacizumab (BCZ)	Anti-VEGF	~149 kDa	Topical, subconjunctival, intrastromal	Low	Cost-effective; widely available; effective in early CoNV	Reduced efficacy in mature vessels; epithelial toxicity with prolonged topical use
Ranibizumab (RBZ)	Anti-VEGF	~48 kDa	Topical, subconjunctival	High	Smaller size → improved tissue penetration; high VEGF-A affinity	No clear superiority over BCZ; short half-life; high cost
Aflibercept (AFL)	Anti-VEGF/PlGF	~115 kDa	Topical, subconjunctival	Very high	Very high VEGF affinity; binds PlGF and PDGF	Mixed efficacy; limited clinical data in CoNV
Brolucizumab	Anti-VEGF	~26 kDa	Not studied in CoNV	Very high	Small size; potent pan-VEGF inhibition	No CoNV data; safety unknown
Faricimab	Anti-VEGF-A/Ang-2	~150 kDa	Not studied in CoNV	Very high	Dual-pathway inhibition	No preclinical or clinical CoNV data
Fine needle diathermy (FND)	Procedural	Thermal occlusion	Intrastromal	Low	Effective for mature, PDGF-stabilised vessels; repeatable	Intrastromal haemorrhage; rare perforation; rebound angiogenesis
Argon laser	Procedural	Energy-based	Stromal	Moderate	Selective haemoglobin absorption	Thermal injury; limited stromal depth
Nd:YAG laser	Procedural	Energy-based	Stromal	Moderate	Effective vessel ablation	Iris damage, haemorrhage; recanalisation
Femtosecond laser	Procedural	Photodisruption	Stromal	Very high	Minimal collateral damage	Limited availability; sparse human data
Photodynamic therapy (PDT)	Procedural	Verteporfin-mediated	Stromal	High	Selective VEC occlusion; minimal tissue injury	Cost; verteporfin shortages; recurrence
Doxycycline/tetracyclines	Adjunctive (anti-MMP)	Small molecule	Oral, topical	Low	MMP inhibition; ↓ IL-1, IL-6, VEGF-C; promotes epithelial healing	Limited clinical evidence; adjunctive role

**Table 4:** Emerging and future therapies for corneal neovascularisation (CoNV): mechanisms, evidence and translational considerations.

THERAPY/AGENT	CLASS	PRIMARY MOLECULAR TARGETS/MECHANISM	ROUTE (S) STUDIED	STAGE OF EVIDENCE	KEY ADVANTAGES	KEY LIMITATIONS/TRANSLATIONAL BARRIERS
Pazopanib	RTKI	VEGFR-1–3, PDGFR, FGFR inhibition	Topical	Early human prospective study	Multi-pathway angiogenic blockade; topical efficacy with minimal systemic exposure	Limited clinical data; no RCTs; systemic toxicity if absorbed
Lapatinib	RTKI	EGFR/HER2 inhibition; ↓ VEGF, MMP activity	Oral (animal)	Preclinical	Inhibits angiogenesis and ECM remodelling	No ocular PK/safety data; unclear translational role
Regorafenib	RTKI	VEGFR, PDGFR, FGFR	Topical (animal)	Preclinical	Broad kinase inhibition	No superiority over BCZ or steroids
Sunitinib	RTKI	VEGFR-2, PDGFR-β, FGFR-1, EGFR	Topical, subconjunctival (animal)	Strong preclinical	Potent dual VEGF/PDGF inhibition; superior to BCZ in models	No human trials; experimental
Axitinib	RTKI	VEGFR, PDGFR; high TIE2 affinity	Topical (animal)	Preclinical	Strong inhibition of angiogenesis	Potential disruption of TIE2 vascular stability signalling
SU6668	RTKI	VEGFR-2, PDGFR-β, FGFR-1 inhibition	Topical (nanoparticle eye drops ± photothermal activation)	Preclinical	Multi-pathway angiogenic inhibition; short-term CoNV suppression in rat models; potential synergy with photothermal vessel occlusion	Evidence limited to animal models
Losartan	Antifibrotic	TGF-β inhibition; ↓ collagen deposition	Topical, systemic	Preclinical + early human	Reduces fibrosis and stromal haze; low toxicity	Indirect anti-angiogenic effect
Decorin	Antifibrotic/gene therapy	TGF-β inhibition; VEGF suppression (context-dependent)	AAV-mediated gene delivery	Preclinical	Endogenous molecule; sustained anti-angiogenic effect	Dual pro/anti-angiogenic roles; gene delivery challenges
Pirfenidone	Antifibrotic	TGF-β modulation; ↓ fibroblast activation	Topical, intravitreal (animal)	Preclinical	Antifibrotic and anti-inflammatory	Limited CoNV-specific data
Nintedanib	RTKI/antifibrotic	VEGFR-2, PDGFR; ↓ MAPK/AKT signalling	Topical, systemic (animal)	Preclinical	Superior to BCZ in models; dual anti-fibrotic/anti-angiogenic	No human trials
Vitamin E	Antioxidant/delivery modifier	ROS scavenging; lipid barrier slows drug diffusion	Topical (lenses, polymers)	Preclinical (alkali burn)	Prolongs ocular residence; enhances bioavailability	Limited direct CoNV endpoints; formulation-dependent
Coenzyme Q10	Antioxidant (mitochondrial)		Topical	Preclinical	Reduced CoNV in inflammatory models; good safety profile	No human CoNV data
Epigallocatechin gallate (EGCG)	Polyphenolic antioxidant	Antioxidant; ↓ VEGF-associated pathways	Topical	Preclinical	Demonstrated CoNV reduction in models	Poor stability; formulation required
Nanoemulsions/microemulsions	Nanodelivery	Enhanced ocular bioavailability	Topical	Preclinical	Improved penetration and sustained release	Formulation complexity; no human data
Nanowafers	Drug delivery platform	Sustained corneal drug release	Topical	Preclinical	Improved compliance; superior to drops in models	Long-term safety and cost unknown
CXL	Physical/biomechanical	↓ angiogenic gene expression; stromal stabilisation	UVA-riboflavin	Early clinical	Neoadjuvant for high-risk grafts; reduces inflammation	HSV reactivation risk; protocol optimisation needed
MICE	Cytotoxic vascular occlusion	DNA cross-linking in neovessels	Intravascular stromal	Early clinical	Targets mature vessels; durable occlusion	Off-licence; limited patient numbers
Aganirsen	Gene-based (antisense)	IRS-1 inhibition → ↓ VEGF	Topical	Phase III	Reduced CoNV; orphan designation	Not commercially available
CRISPR-Cas9	Gene editing	Permanent VEGF-A disruption	Subconjunctival (animal)	Preclinical	Long-term suppression potential	Delivery, off-target effects, immune responses
Viral gene therapy (AAV, lentivirus)	Gene therapy	Endostatin, angiostatin, sVEGFR	Intrastromal	Preclinical	Sustained anti-angiogenic expression	Cost; regulatory and safety barriers
Mesenchymal stem cells (MSCs)	Cell-based therapy	Immunomodulation; ↓ VEGF-C/D	Subconjunctival, intrastromal	Preclinical	Anti-inflammatory and regenerative	Tumorigenicity; standardisation challenges
MSC-derived extracellular vesicles	Cell-free therapy	Delivery of anti-angiogenic mediators	Topical	Preclinical	Lower risk than whole cells	Variable efficacy; early stage
iPSC-derived cells	Regenerative	Restoration of corneal homeostasis	Transplantation	Early clinical (non-CoNV)	Regenerative and immunoregulatory	Tumorigenicity; scalability

## Data Availability

No new data were created or analysed in this study. Data sharing is not applicable to this article.

## Abbreviations

ACAID	anterior chamber–associated immune deviation
AAV	adeno-associated virus
AFL	aflibercept
AIF	apoptosis-inducing factor
α-MSH	alpha-melanocyte stimulating hormone
ANG2/ANGPT2	angiopoietin-2
ANGPT1	angiopoietin-1
ASO	antisense oligonucleotide
bFGF/FGF-2	basic fibroblast growth factor/fibroblast growth factor-2
BCZ	bevacizumab
BM	Bowman’s membrane
CAP	corneal angiogenic privilege
COX-2	cyclooxygenase-2
CoNV	corneal neovascularization
CRISPR	clustered regularly interspaced short palindromic repeats
CsA	cyclosporin A
CXL	corneal collagen cross-linking
ECM	extracellular matrix
EGCG	epigallocatechin gallate
EGFR	epidermal growth factor receptor
ERK	extracellular signal-regulated kinase
FasL	Fas ligand
FGFR	fibroblast growth factor receptor
FND	fine needle diathermy
HIF-1α	hypoxia inducible factor-1 alpha
HER2	human epidermal growth factor receptor-2
HSV-1	herpes simplex virus-1
ICG	indocyanine green (angiography)
IFN-γ	interferon gamma
IK	infectious keratitis
IL	interleukin (e.g., IL-1, IL-6, IL-8)
IPF	idiopathic pulmonary fibrosis
IRS-1	insulin receptor substrate-1
LSCD	limbal stem cell deficiency (concept used; abbreviate if you want)
MAPK	mitogen-activated protein kinase
MCP-1	monocyte chemoattractant protein-1
MICE	mitomycin-C intravascular chemoembolization
miR/miRNA	microRNA
MIF	macrophage migratory inhibitory factor
MMP	matrix metalloproteinase
mTOR	mammalian target of rapamycin
MT1-MMP	membrane type-1 matrix metalloproteinase
Nd:YAG	neodymium:yttrium-aluminium-garnet
NF-κB	nuclear factor kappa-B
NSAID	non-steroidal anti-inflammatory drug
PACK-CXL	photoactivated chromophore for keratitis–corneal cross-linking
PD-L1	programmed death-ligand 1
PDGF	platelet-derived growth factor
PDGFR	platelet-derived growth factor receptor
PI3K	phosphoinositide 3-kinase
PlGF	placental growth factor
PDT	photodynamic therapy
PRK	photorefractive keratectomy
QID	four times daily
RBZ	ranibizumab
RCT	randomised controlled trial
ROS	reactive oxygen species
RSL	refractive laser surgery (appears in one cited title/description; optional)
RTKI/TKI	receptor tyrosine kinase inhibitor/tyrosine kinase inhibitor
sVEGFR1/2	soluble vascular endothelial growth factor receptor-1/-2
TCZ	tocilizumab
TGF-β	transforming growth factor beta
Th17	T helper 17 cell
TIMP	tissue inhibitor of metalloproteinases
TNF-α	tumour necrosis factor alpha
Treg	regulatory T cell
TSP-1/2	thrombospondin-1/-2
UVA	ultraviolet-A
VA	visual acuity
VEC	vascular endothelial cell
VEGF	vascular endothelial growth factor
VEGFR-1/-2/-3	vascular endothelial growth factor receptor-1/-2/-3
VIP	vasoactive intestinal peptide
Wnt/β-catenin	Wnt/beta-catenin signalling pathway
